# Short-term sleep benefits *versus* long-term pulmonary risks: an updated meta-analysis of benzodiazepine receptor positive allosteric modulators in COPD patients with comorbid insomnia

**DOI:** 10.3389/fphar.2026.1636836

**Published:** 2026-04-29

**Authors:** Xiao-Jiao Cui, Bo Xie, Xiao-Qing Yi, Peng-Fei Li

**Affiliations:** 1 Department of Pharmacy, Personalized Drug Research and Therapy Key Laboratory of Sichuan Province, Sichuan Provincial People’s Hospital, University of Electronic Science and Technology of China, Chengdu, China; 2 Chengdu Integrated TCM and Western Medicine Hospital, Chengdu, China; 3 Department of Pharmacy, Bishan Hospital of Chongqing Medical University, Bishan Hospital of Chongqing, Chongqing, China

**Keywords:** benefits, benzodiazepine receptor agonists, COPD, insomnia, risks

## Abstract

**Objective:**

Given the high prevalence and clinical significance of insomnia in COPD patients, and the ongoing controversy surrounding benzodiazepine receptor positive allosteric modulators (PAMs) therapy, this study systematically evaluates the dual-temporal (short- and long-term) safety profile of PAMs through simultaneous assessment of therapeutic benefits and multidimensional risks in COPD-insomnia comorbidity.

**Methods:**

A systematic review and meta-analysis was conducted following the PRISMA guidelines. Systematic literature searches were performed in the target databases. The primary indicators for short-term efficacy and safety assessment comprised total sleep time (TST), number of awakenings, sleep efficiency, partial pressure of oxygen (PaO_2_), oxygen saturation (SaO_2_), FEV_1_, and frequency of apnea events. All-cause mortality and Emergency department, Outpatient, and Hospitalization for acute exacerbations of COPD (AECOPD) were employed as indicators for long-term efficacy and safety.

**Results:**

In COPD patients, PAMs demonstrated significant short-term benefits, with notable improvements in TST (p < 0.00001), frequency of awakenings (p = 0.005), and sleep efficiency (p < 0.00001). Compared with placebo, PAMs did not increase short-term risks in terms of FEV_1_ (p = 0.59), SaO_2_ (p = 0.61), and frequency of apnea events (p = 0.45); however, they induced a slight reduction in PaO_2_ (p = 0.008) during sleep. Notably, long-term use of PAMs was associated with a significant increase in adverse respiratory outcomes, including higher mortality (OR = 1.58, 95% CI: 0.95–2.61, p = 0.08), and elevated frequency of emergency department visits (OR = 1.97, 95% CI: 1.76–2.19, p < 0.00001), outpatient consultations (OR = 2.07, 95% CI: 1.51–2.82, p < 0.00001), and hospitalizations (OR = 1.94, 95% CI: 1.07–3.52, p = 0.03) due to AECOPD.

**Conclusion:**

In patients with COPD and comorbid insomnia, the short-term use of PAMs demonstrates therapeutic potential for improving sleep quality and enhancing quality of life, while not imposing additional respiratory burden. However, prolonged administration may potentially accelerate COPD disease progression. Clinical decisions regarding PAMs therapy for COPD patients with insomnia should be made cautiously after thorough evaluation of short-term benefits *versus* long-term risks.

**Systematic Review Registration:**

https://www.crd.york.ac.uk/PROSPERO/recorddashboard, identifier CRD420251035164.

## 1 Introduction

Chronic Obstructive Pulmonary Disease (COPD) is a heterogeneous pulmonary disorder characterized by persistent and progressive airflow obstruction, which leads to chronic respiratory symptoms including dyspnea, cough, sputum production, and exacerbations (Venkatesan, 2024). COPD ranks among the top three leading causes of mortality worldwide and is characterized by a broad spectrum of comorbidities that can substantially influence disease progression. Insomnia is a prevalent comorbidity among patients with COPD, ranking among the top three complaints reported by this patient population (Budhiraja et al., 2011). The prevalence of insomnia among COPD patients ranges from 24.6% to 47.2%, significantly higher than that in non-COPD individuals (20.3%–25.7%) (Bellia et al., 2003; Budhiraja et al., 2011; Ford et al., 2015), with particularly elevated rates observed in patients with severe COPD and elderly populations (Brewster et al., 2017).

Sleep is critically important for sustaining human health and maintaining vital physiological functions. Importantly, sleep deprivation exerts substantial detrimental effects on disease progression and clinical prognosis in patients with COPD (D’Cruz et al., 2020; Baugh et al., 2022). Shi et al. found that insomnia is an independent risk factor for increased susceptibility to acute exacerbations of COPD (Li et al., 2021). The causal relationship between COPD and insomnia remains unclear. Existing studies have demonstrated that chronic sleep deprivation may induce systemic inflammatory responses (Irwin et al., 2015), hereby accelerating COPD progression and impairing patients’ quality of life (Wedzicha and Seemungal, 2007). Recent research suggests this phenomenon might be associated with specific ligands targeting GABA_A_ receptor subtypes (α4 and α5) (Crocetti and Guerrini, 2020). On the other hand, the primary reasons for the significantly higher prevalence of insomnia in COPD patients compared to healthy populations may include: (1) hypoxemia and hypercapnia caused by COPD (Budhiraja et al., 2015), nocturnal hypoxemia is particularly common in patients with severe COPD, and can trigger microarousing and disrupt normal sleep structures (such as reduced slow-wave sleep and REM sleep). Therefore, we included partial pressure of oxygen, oxygen saturation, and partial pressure of carbon dioxide to assess this pathophysiological dimension. (2) respiratory symptoms inherent to COPD such as persistent cough, sputum production, and increased mucus secretion that impair sleep initiation (Omachi et al., 2012), and (3) the potential respiratory depressant effects of hypnotic medications, particularly benzodiazepine receptor positive allosteric modulators (PAMs) —a drug class that includes traditional benzodiazepines (BZDs) and newer non-benzodiazepine agents (N-BZDs) (Chen et al., 2015). Additionally, factors including tobacco use, COPD therapeutic agents (especially corticosteroids and β_2_-agonists), along with comorbid anxiety and depression, may further disrupt sleep architecture in COPD patients (Budhiraja et al., 2012).

Current therapeutic strategies for improving sleep quality in COPD patients primarily focus on correcting nocturnal hypoxemia through oxygen supplementation or non-invasive ventilation, which may reduce insomnia prevalence in this population (Budhiraja et al., 2012). Pharmacological interventions, particularly hypnotic agents, represent an alternative treatment modality for insomnia management in COPD patients. However, concerns exist regarding the potential of certain hypnotics, especially PAMs, to worsen respiratory function during sleep. Furthermore, accumulating evidence suggests that prolonged use of PAMs may increase the risk of adverse respiratory outcomes in COPD patients (Vozoris et al., 2014; Jiang et al., 2024).

Notably, recent clinical studies have reported no significant adverse effects on respiratory function in COPD patients when administered under controlled conditions (Man et al., 1986; Stege et al., 2010). The suitability of PAMs for sleep improvement in COPD patients remains contentious, with persistent uncertainties regarding their impact on acute exacerbation events and long-term mortality. Despite these findings, considerable controversy persists regarding the appropriateness of PAMs therapy for sleep disturbances in COPD, particularly concerning its effects on acute exacerbation frequency and long-term survival outcomes.

Given the ongoing clinical controversy regarding the respiratory safety of sedative-hypnotics in COPD patients, this systematic review and meta-analysis was conducted with the following pre-specified hypothesis: In patients with COPD and comorbid insomnia, the use of PAMs may provide short-term sleep benefits but is associated with an increased risk of long-term adverse pulmonary outcomes. Accordingly, the primary outcome of this meta-analysis was long-term respiratory safety, operationalized as the incidence of COPD exacerbations and respiratory-related mortality. Secondary outcomes included short-term sleep parameters (e.g., total sleep time, sleep efficiency, and nocturnal awakenings) and changes in pulmonary function tests. This distinction aims to provide a hierarchical evidence synthesis that prioritizes patient safety while acknowledging symptomatic benefits.

## 2 Materials and methods

We rigorously conducted this systematic review in accordance with the Preferred Reporting Items for Systematic Reviews and Meta-Analyses (PRISMA) 2020 guidelines (Supplementary Table S1), (Page et al., 2021) with a prospectively registered protocol (PROSPERO registration number: CRD420251035164).

### 2.1 Data sources and search strategy

A comprehensive literature search was performed in PubMed, Embase, the Cochrane Library, and Web of Science from database inception to 30 March 2025. We used the following search terms and Medical Subject Headings (MeSH), including but not limited to: COPD, insomnia, and benzodiazepine receptor positive allosteric modulators to construct the search strategy. Additionally, to retrieve relevant studies as comprehensively as possible, we also manually searched the reference lists of the aforementioned retrieved articles. We only include studies published in English that involve human subjects. To enhance reproducibility, Supplementary Table S2 provides our detailed search strategy (including PubMed, Embase, the Cochrane Library, and Web of Science).

### 2.2 Study selection

First, two of the authors (CXJ and XB) independently conducted the initial screening of all article titles and abstracts based on the predefined search strategy and inclusion/exclusion criteria, selecting studies that met the eligibility requirements based on their respective judgments. Subsequently, the two authors independently re-examined the full texts of their initially selected studies to assess eligibility criteria, ultimately determining their respective final inclusions through this secondary evaluation process. Finally, the two authors jointly compared and analyzed the studies they each ultimately included. Any discrepancies were resolved through discussion to reach a consensus. When consensus could not be achieved, the third author (LPF) was consulted to adjudicate the disagreements.

### 2.3 Inclusion and exclusion criteria

The inclusion criteria for the meta-analysis were based on population, intervention, comparison, outcome, and study design, as summarized in Table 1. We considered that the study subjects should meet the following criteria: ① having a clear diagnosis of COPD; ② using PAMs as hypnotics, as determined by either a clear study purpose of “improving sleep” or “treating insomnia”, or, when the study purpose was not explicitly stated, a low proportion (<10%) of comorbid anxiety or depression in the study population to maximally exclude off-label use; ③ having obtainable evaluation indicators, such as mortality, blood oxygen saturation, total sleep duration, etc.

**TABLE 1:** Scope of included studies.

Standard	Scope of review
Population	Patients with chronic obstructive pulmonary disease and comorbid insomnia
Intervention	PAMs were used
Comparison	Placebo, comparison between different classes of PAMs
Outcome	Short-term: total sleep time, number of awakenings, sleep efficiency, SaO_2_, PaO_2_, FEV_1_, frequency of apnea events Long-term: all-cause mortality, emergency department, outpatient, and hospitalization for AECOPD
Type of study	RCT, cohort study, case-control study

We excluded ① patients concurrently using opioids such as morphine, as these drugs may independently affect respiratory outcomes; ② COPD patients with post-traumatic stress disorder (PTSD), because PTSD has a unique pathophysiological mechanism and a high rate of psychiatric comorbidity, which may interfere with the assessment of PAMs for insomnia in COPD patients. The presence of PTSD was determined based on explicit reports of the study population in the original studies, baseline comorbidity data of the study population, or the source of the study population (e.g., military personnel, veterans, or refugee cohorts); ③ COPD patients with comorbid obstructive sleep apnea, restless legs syndrome, or a history of circadian rhythm sleep disorders.

### 2.4 Outcomes selection

Total sleep time (TST) is the most direct benefit of hypnotic use in COPD patients, while objective factors such as SaO_2_ and the number of apnea episodes directly impact sleep quality. Therefore, we selected TST, sleep efficiency, and the number of awakenings as indicators to evaluate short-term (≤7 days) benefits, while sleep-period PaO_2_, SaO_2_, FEV_1_, and the number of apnea episodes were used as short-term risk assessment measures. Mortality, a widely accepted key indicator of patient outcomes, serves as the ultimate measure of therapeutic benefit. AECOPD is defined as an acute event characterized by a significant worsening of symptoms, which not only reduces patients’ quality of life but may also accelerate COPD progression, increasing the risk of hospitalization and death (Venkatesan, 2024). Therefore, we selected mortality and the frequency of emergency visits, outpatient consultations, and hospitalizations due to AECOPD (including but not limited to follow-up periods of 30, 60, 90, 180, and 365 days) as long-term (≥30 days) benefit and risk assessment indicators.

### 2.5 Data extraction and processing

The two aforementioned authors independently extracted data from the ultimately included original studies. To ensure data accuracy and consistency, both authors uniformly employed RevMan software for data extraction and organization from the target literature. In accordance with the pre-established study protocol, we extracted the following data: study characteristics (first author, study location, study design, sample size, study purpose); sample characteristics (patient demographics, comorbid conditions at baseline of the study population—particularly those that could bias our meta-analysis results, such as anxiety, depression, and post-traumatic stress—and baseline clinical status including COPD severity and oxygen saturation); intervention details (daily dosage of PAMs, treatment duration); and outcome measures (TST, mortality, total sleep duration, frequency of emergency visits due to COPD exacerbation). Furthermore, the two aforementioned authors examined the purpose of PAM use in each included study on a paper-by-paper basis, and in conjunction with comorbidity information from patient baseline characteristics, only included studies in which the primary treatment goal was insomnia or the proportion of comorbid depression/anxiety was low (<10%).

### 2.6 Quality assessment

Two authors independently assessed the risk of bias in included observational studies using the Newcastle-Ottawa Scale (NOS), while the Cochrane Risk of Bias Tool was employed for RCTs. The NOS is used to assess the risk of bias in cohort studies across three key domains: selection, comparability, and outcome. Each domain is assigned a score, with a maximum total of 9 points. During the evaluation, the two authors may adjust the scores for each domain to reflect the specific characteristics of the study, ensuring that the NOS scale is appropriately tailored for evaluating the included studies. If a study did not explicitly report comorbidities that could potentially affect the meta-analysis results (e.g., depression, anxiety, post-traumatic stress disorder), points were deducted from the comparability score to control for confounding bias. Studies scoring 7–9 are categorized as high quality, 4–6 as moderate quality, and below 4 as low quality (Stang, 2010). Only studies of high quality (scores of 7 or above) were included in our analysis. Additionally, the Cochrane Risk of Bias tool, as recommended by the Cochrane Handbook, evaluates the risk of bias across six domains: selection bias, performance bias, detection bias, attrition bias, reporting bias, and other sources of bias (Martinez et al., 2012).

### 2.7 Heterogeneity, publication bias

Statistical heterogeneity was quantified using the I^2^ statistic. When significant heterogeneity was present (I^2^ > 50%), a random-effects model was employed for heterogeneity analysis. Meta-analysis was performed using RevMan software (version 5.4) with 95% confidence intervals. Additionally, publication bias was visually assessed using funnel plots for the included studies.

### 2.8 Subgroup and sensitivity analyses

We pre-specified three subgroup analyses based on: (1) drug type (benzodiazepines vs. non-benzodiazepines); (2) COPD severity; and (3) duration of use (short-term [<90 days] vs. long-term [≥90 days]). Subgroup analyses were planned when I^2^ was ≥75% and at least two studies were available within a subgroup. In addition, we planned a post-hoc sensitivity analysis by sequentially excluding studies with potential indication confounding and re-performing the meta-analysis to verify the robustness of the main conclusions.

## 3 Results

### 3.1 Search results

**FIGURE 1:**
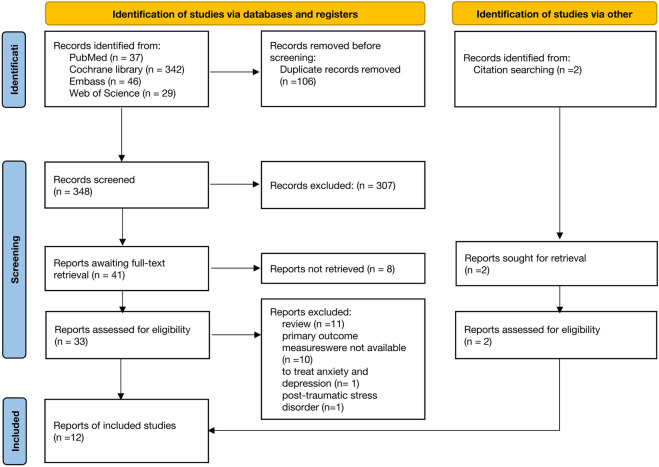
Prisma flow diagram of study inclusion and exclusion.

We conducted a systematic search across four target databases, initially identifying a total of 454 potentially relevant studies. After removing 106 duplicate studies, we subsequently screened the remaining 348 studies based on titles and abstracts, which resulted in 41 candidate studies. Following full-text review and detailed analysis, 10 studies were ultimately selected for inclusion. In addition, we manually screened the reference lists of the retrieved studies, resulting in the inclusion of 2 additional studies. The detailed search strategy is illustrated in Figure 1.

### 3.2 Study characteristics

A total of 12 studies were ultimately included, comprising 7 RCTs, 3 cohort studies, and 2 case-control studies, with a cumulative enrollment of 288,247 patients. The characteristics of the included studies are summarized in Table 2.

**TABLE 2:** Characteristics of the studies included in the meta-analysis.

ID	Study	Region	Patients number	Population	Intervention	Comparison	Outcome	Study design	Objective
1	Stege et al. (2010)	Netherlands	14	Patients with COPD and insomnia	Temazepam (10 mg)	Placebo	PaCO2 during sleep TST; SaO2; desaturation index	RCT	The aims of this study were to assess the effects of temazepam on indices of circadian respiratory function, dyspnea, sleep quality, and sleepiness in patients with severe COPD and insomnia
2	Block (1984)	United States	20	Patients with COPD and insomnia	Flurazepam (30 mg)	Placebo	Frequency of apnea events; episodes of desaturation; TST	RCT	To investigate the effect of flurazepam ingestion on breathing and oxygenation during sleep in patients with COPD.
3	Timms (1988)	United Kingdom	10	Patients with COPD and insomnia	Triazolam (0.125 mg, 0.25 mg)	Placebo	TST; SaO2; the number of apneic and hypopneic events	RCT	To assess the effect of triazolam on sleep and arterial oxygen saturation in patients with COPD.
4	Midgren et al. (1989)	Sweden	14	COPD patients receiving hypnotics	Nitrazepam (5 mg) flunitrazepam (1 mg)	Placebo	TST; SaO2; apneas during sleep	RCT	To quantifying the effects of hypnotics on oxygenation during sleep in patients with stable hypoxemic COPD.
5	Steens et al. (1993)	Canada	23	Patients with COPD and insomnia	Triazolam (0.25 mg) Zolpidem (5 mg, 10 mg)	Placebo	TST; SaO2; the number of awakenings; sleep quality	RCT	Determining the safety and efficacy of novel hypnotics in patients with COPD and insomnia
6	Murciano (1993)	France	12	Hypercapnic COPD patients	Triazolam (0.25 mg) Flunitrazepam (1 mg) Zolpidem (10 mg)	Placebo	FEV_1_; PaO_2_; PaCO_2_	RCT	To compare the hypnotic effects of benzodiazepines in patients with COPD.
7	Wedzicha (1988)	United Kingdom	9	Patients with COPD and insomnia	Diazepam (5 mg)	​	TST; FEV_1_; SaO_2_; number of awakenings	RCT	To investigate the effects of a single dose of diazepam on sleep and respiration in patients with COPD.
8	Chen et al. (2015)	Taiwan	4,868	Patients with COPD and insomnia	Benzodiazepines	No	An increased risk of respiratory failure	Case-control	The aim of this study was to investigate whether the use of BZRAs was associated with an increased risk of respiratory failure in COPD patients
9	Liao et al. (2020)	Taiwan	82,675	The majority of COPD patients in the cohort have insomnia	Benzodiazepines	The patients in the nonuser	Hospitalization, outpatient visits, and emergency visits for AECOPD and all-cause mortality	Cohort study	The aim of our study was to evaluate the drug safety of BZDs in patients with COPD.
10	Chung et al. (2015)	Taiwan	22,684	Patients with COPD and insomnia	Benzodiazepines Non-benzodiazepines	Patients with COPD and no history of adverse respiratory events	Pneumonia, COPD with acute exacerbation, ARF, and cardiopulmonary arrest	Case-control	To evaluate the effects of hypnotics on the risk of adverse respiratory events in patients with COPD.
11	Vozoris et al. (2014)	Canada	177,335	The majority of COPD patients have insomnia	Benzodiazepines	Non-users	Hospitalization, outpatient visits, and emergency visits for AECOPD and all-cause mortality	Cohort study	To evaluate the association of new BZRAs use relative to non-use with adverse clinical respiratory outcomes among older adults with COPD.
12	Jiang et al. (2024)	China	583	Patients with COPD	Benzodiazepines	Non-users	All-cause mortality	Cohort study	To determine whether benzodiazepine use is associated with increased all-cause mortality in patients with COPD.

### 3.3 Short-term benefits assessment

#### 3.3.1 TST

**FIGURE 2:**
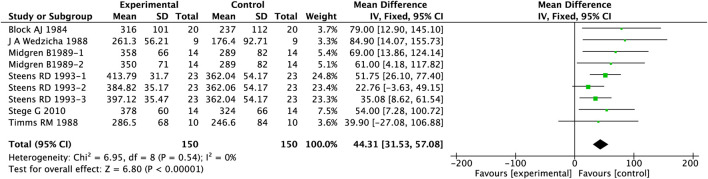
Effect of PAMs on TST in COPD patients with comorbid insomnia.

In the cohort analysis evaluating the impact of PAMs on TST in COPD patients with insomnia, 6 studies involving 300 patients were included. Given the low heterogeneity observed in this cohort (I^2^ = 0%), a fixed-effects model was adopted. Detailed results of PAMs’ effects on TST are illustrated in Figure 2. Our meta-analysis demonstrated that PAMs significantly prolonged TST in this patient population (MD = 44.31, 95%CI: 31.53–57.08, p < 0.00001).

#### 3.3.2 Number of awakenings

**FIGURE 3:**
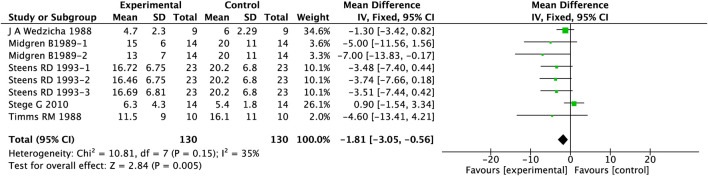
Effect of PAMs on number of awakenings in COPD patients with comorbid insomnia.

In the analysis assessing the effect of PAMs on number of awakenings in COPD patients with comorbid insomnia, 5 studies involving 260 patients were included. A fixed-effects model was applied based on low heterogeneity (I^2^ = 35%), and detailed outcomes are presented in Figure 3. The results demonstrated a significant reduction in nighttime awakenings with PAMs compared to placebo (MD = −1.18, 95%CI: 3.05 to −0.56, p = 0.005).

#### 3.3.3 Sleep efficiency

**FIGURE 4:**
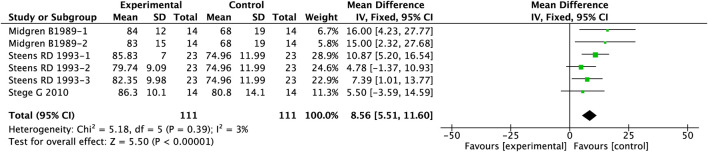
Effect of PAMs on sleep efficiency in COPD patients with comorbid insomnia.

This cohort incorporated three studies involving 222 patients, with sleep efficiency quantitatively assessed using the Visual Analogue Scale and Digit Symbol Substitution Test. The detailed analytical findings are presented in Figure 4. Compared to placebo, PAMs demonstrated significant improvement in sleep efficiency among COPD patients (MD = 8.56, 95%CI: 5.51–11.60, p < 0.00001).

### 3.4 Short-term risks assessment

#### 3.4.1 PaO_2_

**FIGURE 5:**
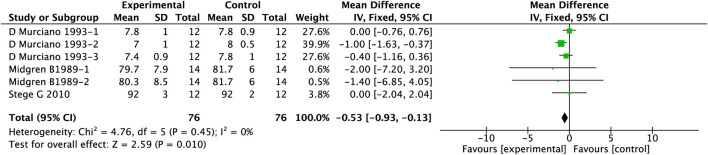
Effect of PAMs on PaO2 in COPD patients with comorbid insomnia.

PAMs exhibit known respiratory depressant effects. Our meta-analysis incorporated data from 3 clinical studies involving 152 patients with COPD and comorbid insomnia. The detailed analytical results are presented in Figure 5. Our findings demonstrated a significant reduction in nocturnal PaO_2_ levels during sleep in COPD patients receiving PAMs compared to controls (MD = −0.53, 95%CI: 0.93 to −0.13, p = 0.010).

#### 3.4.2 FEV_1_

**FIGURE 6:**
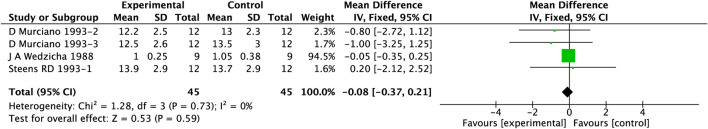
Effect of PAMs on FEV_1_ in COPD patients with comorbid insomnia.

FEV_1_, as one of the most widely utilized parameters for evaluating pulmonary ventilation function in COPD patients, is universally recognized. The cohort investigating the effects of PAMs on FEV_1_ incorporated two studies involving 90 patients. Detailed analytical outcomes are delineated in Figure 6. Our meta-analysis demonstrated no statistically significant impact of PAMs on pulmonary ventilation function in COPD patients (MD = −0.08, 95%CI: 0.37– 0.21, p = 0.59).

#### 3.4.3 Frequency of apnea events

**FIGURE 7:**
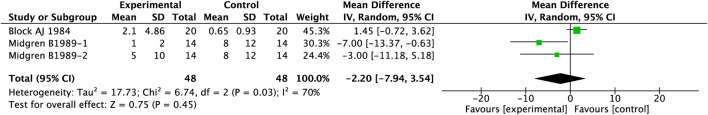
Effect of PAMs on frequency of apnea events in COPD patients with comorbid insomnia.

Apnea is another important indicator for evaluating the inhibitory effects of hypnotics on nocturnal respiration in COPD patients. This research cohort included two studies involving 96 COPD patients with insomnia. Due to significant heterogeneity (I^2^ = 70%), a random-effects model was adopted. The analysis of PAMs’ impact on sleep apnea in COPD patients, as shown in Figure 7, demonstrated no significant effect compared to placebo (MD = −2.20, 95%CI: 7.94– 3.54, p = 0.45).

#### 3.4.4 SaO_2_

**FIGURE 8:**
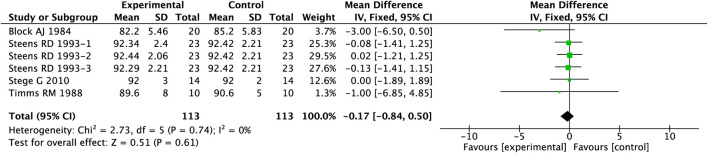
Effect of PAMs on SaO_2_ in COPD patients with comorbid insomnia.

The primary mechanism underlying insomnia in COPD patients is attributed to arousal responses induced by nocturnal oxygen desaturation. Therefore, changes in SaO_2_ serve as the fundamental metric for assessing the impact of hypnotic agents on sleep quality in this population. Our meta-analysis incorporated data from 4 clinical studies involving 226 patients with COPD and comorbid insomnia. As detailed in Figure 8, the analysis using a fixed-effects model revealed that PAMs demonstrated no statistically significant effect on nocturnal SaO_2_ levels compared to placebo (MD = −0.17, 95%CI: 0.84– 0.50, p = 0.61).

### 3.5 Long-term benefit-risk assessment

#### 3.5.1 All-cause mortality

**FIGURE 9:**
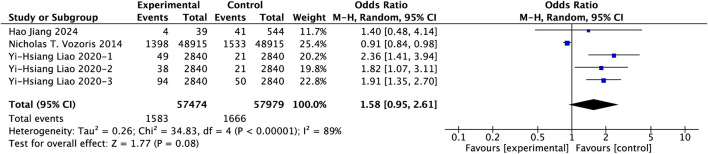
Effect of PAMs on all-cause mortality in COPD patients with comorbid insomnia.

All-cause mortality, as a universally accepted endpoint for evaluating ultimate patient benefit, was analyzed across a pooled cohort of three large-scale observational studies involving 115,453 COPD patients. Due to significant heterogeneity (I^2^ = 89%), a random-effects model was adopted. Figure 9 presents the detailed impact of PAMs on mortality risk in COPD patients. The results of our study suggest that long-term use of PAM is associated with a potentially increase in mortality risk, approaching the threshold for statistical significance (OR = 1.58, 95% CI: 0.95–2.61, p = 0.08).

#### 3.5.2 Outpatient for AECOPD

**FIGURE 10:**
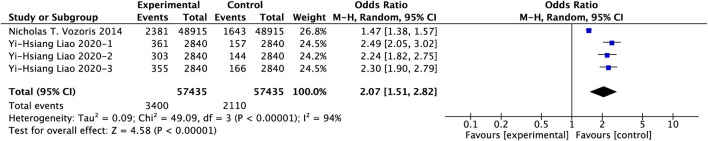
Effect of PAMs on outpatient for AECOPD in COPD patients with comorbid insomnia.

Outpatient for AECOPD, serving as an objective proxy for COPD disease progression, was evaluated across two population-based cohort studies encompassing 114,870 patients. Substantial methodological heterogeneity (I^2^ = 94%) prompted utilization of a random-effects model. As shown in Figure 10. After adjusting for potential confounders, PAMs exposure was associated with a 107% higher likelihood of AECOPD-related healthcare encounters (OR = 2.07, 95% CI: 1.51–2.82, p < 0.00001) in propensity score-matched analyses.

#### 3.5.3 Hospitalization for AECOPD

**FIGURE 11:**
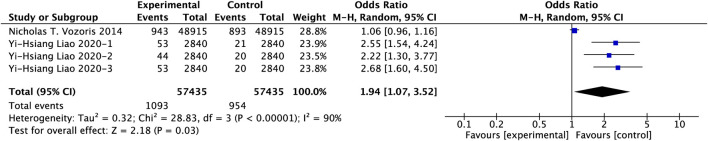
Effect of PAMs on hospitalization for AECOPD in COPD patients with comorbid insomnia.

The number of AECOPD-related hospitalizations, serving as another objective measure of COPD severity, was analyzed across two studies comprising 114,870 patients. Using random-effects models (I^2^ = 90%), our meta-analysis revealed that PAMs use was significantly associated with increased hospitalization rates (OR = 1.94, 95% CI: 1.07–3.52, p = 0.03), as detailed in Figure 11.

#### 3.5.4 Emergency department for AECOPD

**FIGURE 12:**
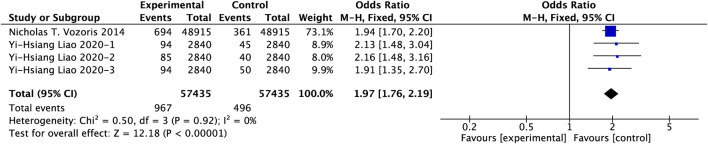
Effect of PAMs on emergency department for AECOPD in COPD patients with comorbid insomnia.

Our analysis incorporated two large-scale studies comprising 114,870 COPD patients. Using fixed-effects models, we found that PAMs hypnotic use was independently associated with increased AECOPD-related emergency department visits (OR = 1.97, 95% CI: 1.76–2.19, p < 0.00001), as detailed in Figure 12.

### 3.6 Subgroup and sensitivity analyses

In accordance with pre-specified analyses, subgroup analyses were performed by (1) drug type (benzodiazepines [BZDs] vs. non-benzodiazepines [N-BZDs] vs. mixed use). Results for mortality, and for hospitalizations, outpatient visits, and emergency department visits due to acute exacerbations of COPD (AECOPD), are presented in Supplementary Figures S2–S5, respectively.

For all-cause mortality, significant associations were observed for N-BZDs (OR = 1.82, 95% CI: 1.07–3.11) and mixed use (OR = 1.91, 95% CI: 1.35–2.70), but not for BZDs alone (OR = 1.40, 95% CI: 0.67–2.67). For AECOPD-related outpatient visits, risks were higher for N-BZDs (OR = 2.24, 95% CI: 1.82–2.75) and mixed use (OR = 3.35, 95% CI: 2.70–4.17) compared to BZDs alone (OR = 1.90, 95% CI: 1.13–3.17). For AECOPD-related hospitalizations, N-BZDs (OR = 2.22, 95% CI: 1.30–3.77) and mixed use (OR = 2.68, 95% CI: 1.60–4.50) again showed higher risks, compared to BZDs alone (OR = 1.09, 95% CI: 1.00–1.20). These findings should be interpreted cautiously due to high subgroup heterogeneity and the inclusion of only one study each in the N-BZD and mixed-use groups.

Due to insufficient data, pre-specified subgroup analyses for (2) COPD severity and (3) duration of use (short-term [<90 days] vs. long-term [≥90 days]) could not be performed. Regarding COPD severity, one non-pooled study found that among patients with no prior-year AECOPD, BZD use was associated with increased hospitalization for COPD/pneumonia (RR = 1.29, 95% CI: 1.07–1.56); this association was not observed in patients with ≥1 prior AECOPD (Vozoris et al., 2013). Regarding duration of use, another non-pooled study reported that short-term BZD use was associated with higher mortality (HR = 1.16, 95% CI: 1.05–1.28) (Donovan et al., 2019).

The results of the leave-one-out sensitivity analysis showed that the findings for the vast majority of outcome measures remained relatively stable. The sensitivity analysis results for each outcome measure are presented in Supplementary Figures S6–S16.

### 3.7 Risk of bias assessment

**FIGURE 13:**
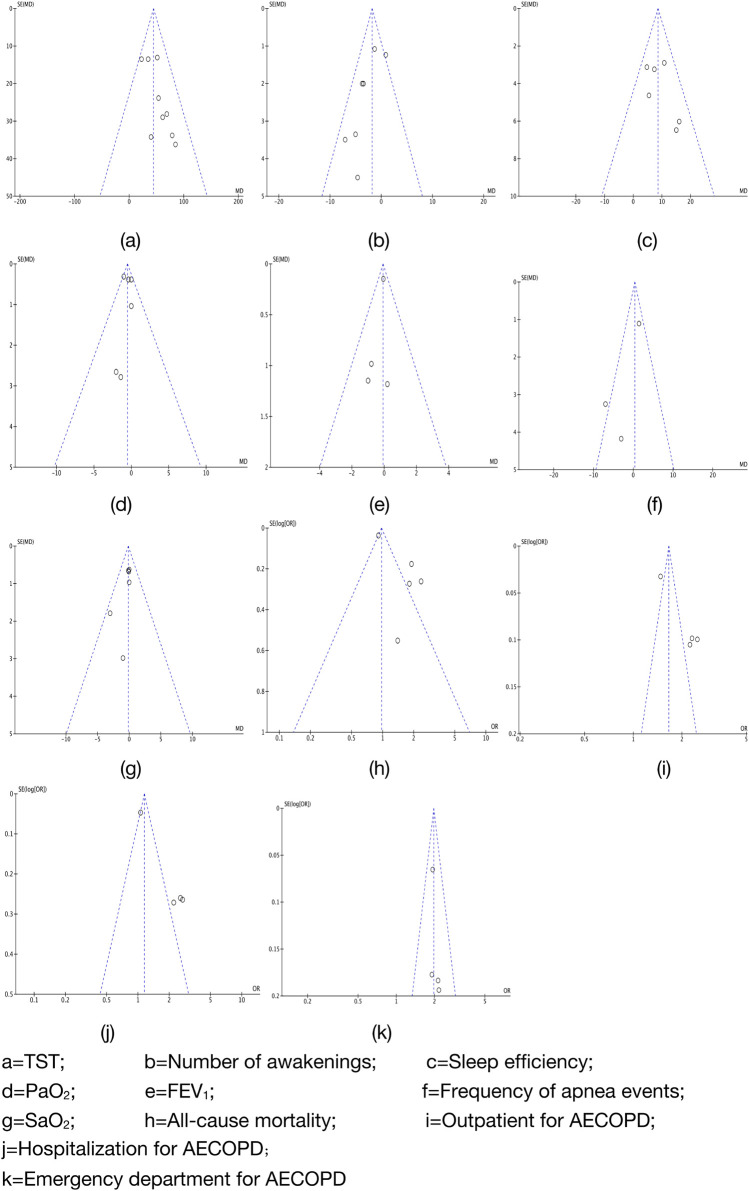
Funnel plots for publication bias assessment. **(a)** TST; **(b)** number of awakenings; **(c)** sleep efficiency; **(d)** PaO_2_; **(e)** FEV_1_; **(f)** frequency of apnea events; **(g)** SaO_2_; **(h)** all-cause mortality; **(i)** outpatient for AECOPD; **(j)** hospitalization for AECOPD; **(k)** emergency department for AECOPD. Each plot shows individual study results (points) within a triangle formed by dashed lines, illustrating the spread of standard error and effect size or odds ratio.

The risk of bias assessment table for observational studies can be found in Supplementary Table S2, and the risk of bias assessment graph for RCT studies is provided in Supplementary Figure S1. Additionally, we visually evaluated publication bias using funnel plots, and no significant publication bias was detected. Detailed results are shown in Figure 13.

## 4 Discussion

Insomnia is prevalent among COPD patients, and inadequate treatment of insomnia may increase the risk of adverse respiratory outcomes in COPD patients (Midgren et al., 1989; Lu et al., 2016). PAMs are currently the most commonly used approach to improve sleep in COPD patients (Halvorsen and Martinussen, 2015). Pharmacodynamic studies indicate that they exert hypnotic effects by binding BZDs or N-BZDs to different subunits of the GABA_A_ receptor (Gravielle, 2016). Although earlier studies have investigated the benefits and risks of PAMs in COPD patients, the findings remain inconclusive, with a predominant focus on short-term respiratory effects (Midgren et al., 1989; Murciano et al., 1993; Mandrioli et al., 2010). A previous meta-analysis by (Lu et al., 2016) demonstrated that PAMs might be effective and safe for managing insomnia in patients with COPD, based on evaluations of total sleep time, sleep efficiency, sleep latency, and awakenings per hour. In recent years, several large population-based observational studies have investigated respiratory-related adverse events associated with long-term PAMs use in COPD patients (Vozoris et al., 2014; Jiang et al., 2024). Our study expands upon these findings by incorporating critical pulmonary function parameters (PaO_2_, FEV_1_, and SaO_2_) and specifically assessing the long-term safety of PAMs in COPD patients. Given the chronic nature of sleep disturbances in this population, prolonged PAMs use is often clinically warranted. We evaluated long-term safety outcomes, including all-cause mortality AECOPD and rates of emergency visits, hospitalizations, and outpatient admissions. Our results suggest that while short-term PAMs use may improve sleep quality, prolonged administration could potentially accelerate COPD progression. To the best of our knowledge, this represents the first investigation to address this clinical question through dual evaluation of short-term and long-term impacts. Furthermore, to enhance the reliability of our findings, we exclusively included RCTs for the short-term benefit-risk assessment, while for the long-term evaluation, we only incorporated large population-based observational studies. Additionally, we conducted bias risk assessments for both the RCTs and observational studies separately.

Regarding short-term benefits and risks, findings from several previous double-blind, randomized, placebo-controlled, crossover studies demonstrated that PAMs significantly improved sleep outcomes—including TST, number of awakenings, and sleep efficiency—without exacerbating the deterioration of patients’ pulmonary function (Murciano et al., 1990; Steens et al., 1993; Stege et al., 2010). Short-term use of PAMs in COPD patients with insomnia is effective and safe, which is consistent with our study findings. However, while our meta-analysis demonstrated statistically significant improvements in objective sleep parameters such as total sleep time and sleep efficiency, the clinical meaningfulness of these findings warrants critical consideration. It remains uncertain whether these modest improvements translate into tangible benefits in patient-reported outcomes (PROs), such as subjective sleep quality, daytime functioning, or health-related quality of life. Previous research in patients with chronic insomnia has highlighted frequent discrepancies between objective polysomnographic measures and patient perceptions of sleep (Bianchi et al., 2013; Masaki et al., 2025). In the context of COPD, where sleep disruption is often multifactorial—influenced by dyspnea, cough, and anxiety—the incremental value of pharmacologically induced sleep over non-pharmacological interventions requires careful weighing against potential risks. Given this uncertainty, future studies should prioritize the inclusion of validated PRO instruments to ensure that therapeutic interventions align with what matters most to patients.

Regarding safety considerations, the respiratory effects of PAMs typically result from frequent administration or misuse (Kozole Smid et al., 2024). In our analysis, all included studies employed standard or even low therapeutic doses. As the most commonly prescribed BZDs hypnotic, alprazolam has a wide therapeutic window, with toxic concentrations generally exceeding 100–400 ng/mL - far above the levels achieved at recommended hypnotic doses (Shah et al., 2012; Ojanperä et al., 2016). Kuzniar and colleagues similarly found that respiratory depression and brainstem reflex impairment occur only with zolpidem (an N-BZDs hypnotic) overdose (Marrache et al., 2004). The study by Stege et al. demonstrated that temazepam improved sleep quality in patients with severe COPD without significantly affecting respiratory parameters during sleep, including SaO_2_ and partial pressure of carbon dioxide. Notably, this study employed a relatively low temazepam dose (10 mg); different outcomes might potentially occur with higher doses (20 mg). However, these studies assessed only single or short-term dosing effects, contrasting with the chronic prescribing patterns of hypnotics in real-world settings.

Beyond dose-related safety, our findings also revealed subtle changes in gas exchange. Specifically, our findings indicate that short-term PAMs use in COPD patients may lead to decreased PaO_2_ during sleep, while no significant alterations were observed in SaO_2_, apnea events, or FEV_1_. This discrepancy may be attributed to PaO_2_ being more sensitive than SaO_2_ in detecting hypoxic changes. At lower therapeutic doses, PAMs typically induce only minor modifications in pulmonary gas exchange. These subtle changes can promptly affect PaO_2_ levels, whereas SaO_2_ variations become more apparent only under more pronounced hypoxic conditions (Pruitt, 2024). A certain degree of hypoventilation and a decrease in PaO_2_ from wakefulness to sleep are physiological processes. A mild decrease in PaO_2_ typically does not exacerbate insomnia in stable COPD patients, nor does it necessitate nocturnal oxygen therapy (Budhiraja et al., 2015). Several mechanisms contribute to nocturnal oxygen desaturation in COPD. The primary mechanism is likely alveolar hypoventilation, which reduces minute ventilation. During sleep, chemoreceptors and the ventilation center become the primary regulators of respiration, leading to a PaO_2_ decrease of 3–10 mmHg (Becker et al., 1999). In individuals with stable COPD, who generally have high oxygen reserves, this mild decrease may result in only a slight reduction in blood oxygen saturation. However, during COPD exacerbations, when SaO_2_≤93%, indicating limited oxygen reserves, even a small decline in PaO_2_ may lead to a significant drop in oxygen saturation. This is particularly concerning as it occurs within the steep portion of the oxyhemoglobin dissociation curve, where small changes in PaO_2_ correspond to substantial changes in oxygen saturation. Daytime SaO_2_ is a strong predictor of nocturnal oxygen desaturation in COPD patients (Mulloy et al., 1995). Furthermore, medical guidelines mandate nocturnal oxygen therapy when arterial PaO_2_ drops to ≤55 mmHg or oxygen saturation falls to ≤88% for at least 5 min during sleep. A significant decrease in PaO_2_—more than 10 mmHg, or a drop in oxygen saturation exceeding 5% over at least 5 minutes—can accelerate the onset of insomnia (Ramsey et al., 2007). Significant nocturnal PaO_2_ desaturation in COPD patients can also increase systemic systolic blood pressure and mean pulmonary artery pressure, which in turn leads to higher healthcare utilization, particularly hospital admissions due to COPD exacerbations (Sajkov and McEvoy, 2009). Additionally, it may contribute to elevated nighttime mortality rates in this patient population (Lacasse et al., 2011). In line with Midgren and colleagues’ results, the study found that hypnotics did not influence carbon dioxide partial pressure in COPD patients (Midgren et al., 1989). This phenomenon may be attributed to carbon dioxide’s superior diffusion capacity compared to oxygen, whereby minor ventilation impairment fails to significantly alter PaCO_2_ levels in COPD patients.

Regarding long-term benefits and risks, our meta-analysis identified a significant association between long-term PAMs use and all-cause mortality in COPD patients, along with elevated risks of emergency department visits, outpatient visits, and hospitalizations for AECOPD. However, the interpretation of this finding must be tempered by the potential for confounding by indication, a key limitation of the included observational studies. This bias arises because clinicians are more likely to prescribe sedative-hypnotics to patients with more severe insomnia or a higher burden of comorbid conditions. For instance, Vozoris et al. suggested that observed associations in such studies might be an artifact of selective prescribing patterns (Vozoris et al., 2013). Although some included studies attempted to mitigate this bias by adjusting for measured covariates such as age, sex, and comorbidities (Vozoris et al., 2013; Chen et al., 2015; Liao et al., 2020), residual confounding by unmeasured or imperfectly measured factors—including frailty, untreated depression, poor social support, or specific sleep disorder characteristics—cannot be excluded. Therefore, while our findings raise concern regarding the long-term safety of PAMs in this population, they should not be interpreted as evidence of direct causation. Rather, they should be viewed as associations that may reflect both the pharmacological effects of the medication and the underlying risk profile of the patients prescribed them. This interpretation underscores the need for careful patient selection and monitoring when considering long-term PAM therapy, as well as the urgent need for well-designed prospective studies with rigorous confounding control to disentangle these effects.

These findings are largely consistent with existing studies, supporting that long-term use of hypnotic drugs may further worsen the condition of COPD patients. This may be related to factors such as drug tolerance from prolonged and frequent use of hypnotics (Hata et al., 2018; DeKosky and Williamson, 2020). An evaluative study assessed the latest evidence on short-term and long-term use of PAMs through three approaches: a national survey, a review of current clinical and preclinical studies, and statements from an expert panel on insomnia. The results demonstrated that short-term use (≤3 weeks) of hypnotics is safe and effective, while long-term use carries potential risks. It was recommended that risks be carefully reevaluated before opting for prolonged therapy (Zee et al., 2023). The challenges of managing chronic insomnia demonstrate that, in real-world practice, it is difficult to prevent short-term use from evolving into long-term use. Many PAMs prescriptions are used for unauthorized or extended periods (typically 2 weeks for sleeping pills) (Lader, 2011). On the one hand, patients find these medications helpful, have no unbearable side effects, and their effectiveness does not diminish over time. They are generally reluctant to stop using them (Cook et al., 2007). On the other hand, with the increasing availability of prescription drugs online, the opportunity to obtain PAMs without a prescription may increase in many countries. The report states that PAMs are the most frequently offered regulated drugs online, with an estimated 89% of online supply websites allowing purchase without a doctor’s prescription (Levine, 2007). Therefore, while our meta-analysis may demonstrate efficacy for short-term use, the real-world propensity for dependency necessitates a cautious interpretation of these benefits. This evidence strongly reinforces the imperative that any initiation of PAMs therapy for insomnia must be accompanied by strict adherence to prescribing guidelines, patient education on risks, and a clear plan for discontinuation from the outset.

Current research suggests that the efficacy and safety of PAMs in COPD patients with insomnia may be influenced by multiple factors, including drug type, dosage, and psychological conditions (e.g., anxiety/depression), but are likely unrelated to drug half-life or disease severity. Regarding pharmacological class (Chen et al., 2015) reported differential risks of respiratory failure among COPD patients using various hypnotic regimens. Their findings revealed that while BZDs monotherapy (OR = 1.58, 95% CI: 1.14–2.20) and combination therapy (OR = 2.25, 95% CI: 1.48–3.43) were both significantly associated with increased respiratory failure risk compared to non-users, N-BZDs monotherapy demonstrated no significant risk elevation (OR = 0.85, 95% CI: 0.51–1.44).

A potential explanation is that, unlike BZDs, N-BZDs exhibit higher selectivity for specific benzodiazepine receptor subtypes (primarily the ω1 subunit of GABA-A receptors) in the central nervous system, resulting in fewer adverse effects on pulmonary function (Vozoris et al., 2013). Regarding dosage effects, a nationwide prospective cohort study from Sweden revealed a dose-dependent trend between higher PAMs dosage and increased mortality (OR = 1.01, 95% CI: 1.00–1.03) (Ekström et al., 2014). Additionally, Steens et al. found that zolpidem 10 mg was superior to zolpidem 5 mg in terms of both sleep quality and sleep onset latency (Steens et al., 1993), with similar results observed in studies of healthy populations (Merlotti et al., 1989). Regarding elimination half-life, the third sensitivity analysis in Liao and colleagues’ study stratified PAMs by their half-lives, revealing no significant differences in respiratory outcomes among COPD patients across different half-life groups (Liao et al., 2020). This may be because hypnotics are typically administered once daily at low doses, preventing systemic accumulation—despite the fact that most PAMs metabolites have longer half-lives than their parent compounds. Regarding disease severity, Tinedo et al. found no association between PAMs use and either readmission rates or mortality in COPD patients, regardless of COPD severity level (Tinedo et al., 2018). Similarly, Vozoris and colleagues found consistent results across all COPD severity subgroups regarding increased outpatient and emergency department visits due to AECOPD. However, for AECOPD-related hospitalizations and ICU admissions, significant differences were observed between PAMs users and non-users in the least severe subgroup, while no significant differences were found in more severe subgroups. This discrepancy may be attributable to non-patient-related factors that could not be adjusted for in the analysis (e.g., hospital bed availability and physician judgment) (Vozoris et al., 2014). Notably, the joint American Thoracic Society/European Respiratory Society guidelines recommend cautious use of PAMs in severe COPD patients without providing specific rationale or evidenc (Qaseem et al., 2011). Our research was originally intended to investigate the relationship between the severity of COPD and the risk-benefit of using PAMs. Unfortunately, we were unable to obtain pulmonary function data from the included original studies and thus could not conduct further analysis.

Beyond these clinical and pharmacological factors, methodological heterogeneity in outcome measurement and definition likely contributed to the observed variability. For short-term outcomes, the included studies employed different measurement methods and time points for assessing total sleep time (TST), number of awakenings, sleep efficiency, and respiratory parameters such as PaO_2_, SaO_2_, FEV_1_, and apnea events. These methodological differences may have introduced variability that could not be fully accounted for in pooled analyses. For long-term outcomes, definitions of all-cause mortality and AECOPD-related events (emergency department visits, outpatient visits, and hospitalizations) varied across studies, with follow-up periods ranging from 30 to 365 days. Such differences in outcome ascertainment and duration of follow-up likely contributed to the substantial heterogeneity observed in safety outcomes.

European guidelines for the diagnosis and treatment of insomnia recommend cognitive behavioral therapy (CBT-I) as the first-line treatment for chronic insomnia in adults (Riemann et al., 2023), a recommendation that closely mirrors those of the American Academy of Sleep Medicine (Sateia et al., 2017). However, several significant barriers to the implementation of CBT-I for insomnia persist in clinical practice. On a personal level, insomnia is often stigmatized, with many individuals perceiving it as a trivial issue rather than a legitimate clinical concern, leading to feelings of being misunderstood by the medical community. At the clinician level, there is a widespread lack of knowledge about treatment options like CBT-I, and few healthcare providers are adequately trained to deliver CBT-I for insomnia (Araújo et al., 2017). Moreover, a systematic review of ten studies on sleep disorders found that seven demonstrated significant benefits of CBT-I over placebo, including improvements in sleep efficiency, sleep quality, and total sleep time (Salame et al., 2025). In cases where CBT-I is ineffective or unavailable, pharmacotherapy may be considered as an alternative treatment.

Although we strictly followed the guidelines for systematic reviews in conducting this meta-analysis, our study has several limitations. First, the included studies comprised both randomized controlled trials and observational studies, with varying research backgrounds and objectives, contributing to substantial heterogeneity across outcomes. This heterogeneity likely arose from differences in study design, patient populations (e.g., COPD severity), intervention characteristics (drug type and dosage), and outcome measurement methods. Moreover, although we attempted to control for indication bias through our inclusion criteria and sensitivity analyses, the possibility that PAMs were used for non-insomnia purposes in some studies cannot be completely excluded, which may represent a potential source of bias in this study. In addition, asymmetry was found upon visual inspection of the funnel plots of these outcomes (funnel plots j and k), suggesting possible publication bias or other small-sample effects that may have further exacerbated the observed heterogeneity and exaggerated the overall effect estimate. Second, regarding long-term benefit-risk assessment, the follow-up periods across the studies were inconsistent. Finally, the retrospective nature of most included studies limited control for potential confounders such as oxygen therapy and medication adherence.

## 5 Conclusion

Short-term use of PAMs in COPD patients with insomnia is effective and safe, significantly improving sleep quality and overall wellbeing without worsening respiratory burden. However, long-term PAMs use may accelerate COPD progression and significantly increase the risk of adverse respiratory outcomes. Therefore, long-term PAMs therapy is not recommended for routine sleep management in COPD patients due to the potential risks outweighing the benefits. Clinical decision-making should be individualized, considering factors such as disease severity, patient preferences, life expectancy (e.g., in terminal care), and access to alternative therapies.

## Data Availability

The original contributions presented in the study are included in the article/Supplementary Material, further inquiries can be directed to the corresponding author.
