# Supplementary material for: Short-term sleep benefits versus long-term pulmonary risks: an updated meta-analysis of benzodiazepine receptor positive allosteric modulators in COPD patients with comorbid insomnia
Source: Front Pharmacol. 2026 Apr 29;17:1636836. doi: 10.3389/fphar.2026.1636836 (PMC13167996; doi:10.3389/fphar.2026.1636836)

Appendix 1. Prisma checklist

Table S1. Prisma checklist

Section/topic	#	Checklist item
**TITLE**		
Title	1	Identify the report as a systematic review, meta-analysis, or both.
**ABSTRACT**		
Structured summary	2	Provide a structured summary including, as applicable: background; objectives; data sources; study eligibility criteria, participants, and interventions; study appraisal and synthesis methods; results; limitations; conclusions and implications of key findings; systematic review registration number.
**INTRODUCTION**		
Rationale	3	Describe the rationale for the review in the context of what is already known.
Objectives	4	Provide an explicit statement of questions being addressed with reference to participants, interventions, comparisons, outcomes, and study design (PICOS).
**METHODS**		
Protocol and registration	5	Indicate if a review protocol exists, if and where it can be accessed (e.g., Web address), and, if available, provide registration information including registration number.
Eligibility criteria	6	Specify study characteristics (e.g., PICOS, length of follow-up) and report characteristics (e.g., years considered, language, publication status) used as criteria for eligibility, giving rationale.
Information sources	7	Describe all information sources (e.g., databases with dates of coverage, contact with study authors to identify additional studies) in the search and date last searched.
Search	8	Present full electronic search strategy for at least one database, including any limits used, such that it could be repeated.

**Study selection**	**9**	**State the process for selecting studies (i.e., screening, eligibility, included in systematic review, and, if applicable, included in the meta-analysis).**
**Data collection process**	**10**	**Describe method of data extraction from reports (e.g., piloted forms, independently, in duplicate) and any processes for obtaining and confirming data from investigators.**
**Data items**	**11**	**List and define all variables for which data were sought (e.g., PICOS, funding sources) and any assumptions and simplifications made.**
**Risk of bias in individual studies**	**12**	**Describe methods used for assessing risk of bias of individual studies (including specification of whether this was done at the study or outcome level), and how this information is to be used in any data synthesis.**
**Summary measures**	**13**	**State the principal summary measures (e.g., risk ratio, difference in means).**
**Synthesis of results**	**14**	**Describe the methods of handling data and combining results of studies, if done, including measures of consistency (e.g., I^2) for each meta-analysis.**

**Section/topic**	**#**	**Checklist item**
**Risk of bias across studies**	**15**	**Specify any assessment of risk of bias that may affect the cumulative evidence (e.g., publication bias, selective reporting within studies).**
**Additional analyses**	**16**	**Describe methods of additional analyses (e.g., sensitivity or subgroup analyses, meta-regression), if done, indicating which were pre-specified.**
**RESULTS**		
**Study selection**	**17**	**Give numbers of studies screened, assessed for eligibility, and included in the review, with reasons for exclusions at each stage, ideally with a flow diagram.**
**Study characteristics**	**18**	**For each study, present characteristics for which data were extracted (e.g., study size, PICOS, follow-up period) and provide the citations.**

**Risk of bias within studies**	**19**	**Present data on risk of bias of each study and, if available, any outcome level assessment (see item 12).**
**Results of individual studies**	**20**	**For all outcomes considered (benefits or harms), present, for each study: (a) simple summary data for each intervention group (b) effect estimates and confidence intervals, ideally with a forest plot.**
**Synthesis of results**	**21**	**Present results of each meta-analysis done, including confidence intervals and measures of consistency.**
**Risk of bias across studies**	**22**	**Present results of any assessment of risk of bias across studies (see Item 15).**
**Additional analysis**	**23**	**Give results of additional analyses, if done (e.g., sensitivity or subgroup analyses, meta-regression [see Item 16]).**
**DISCUSSION**		
**Summary of evidence**	**24**	**Summarize the main findings including the strength of evidence for each main outcome; consider their relevance to key groups (e.g., healthcare providers, users, and policy makers).**
**Limitations**	**25**	**Discuss limitations at study and outcome level (e.g., risk of bias), and at review-level (e.g., incomplete retrieval of identified research, reporting bias).**
**Conclusions**	**26**	**Provide a general interpretation of the results in the context of other evidence, and implications for future research.**
**FUNDING**		
**Funding**	**27**	**Describe sources of funding for the systematic review and other support (e.g., supply of data); role of funders for the systematic review.**

Appendix 2. Search strategie

Table S2. Search strategy

PUBMED		
**Search number**	**Query**	**Results**
14	#4 and #8 and #13	37
13	#9 or #10 or #11 or #12	90,134
12	BZRA	80
11	BZD	1,376
10	benzodiazepines	89,989
9	benzodiazepines[MeSH Terms]	71,530
8	#5 or #6 or #7	145,698
7	Sleep disturbances	130,176
6	insomnia	40,066
5	insomnia[MeSH Terms]	19,911
4	#1 or #2 or #3	116,072
3	COPD	116,072
2	chronic obstructive pulmonary disease	106,142
1	chronic obstructive pulmonary disease[MeSH Terms]	71,742
**Cochrane library**		
#1	MeSH descriptor: [Pulmonary Disease, Chronic Obstructive] explode all trees	7976
#2	(chronic obstructive pulmonary disease):ti,ab,kw OR (COPD):ti,ab,kw	24003
#3	(BZD):ti,ab,kw OR (BZRA):ti,ab,kw OR (benzodiazepines):ti,ab,kw	5076
#4	MeSH descriptor: [Benzodiazepines] explode all trees	11686
#5	MeSH descriptor: [Sleep Initiation and Maintenance Disorders] explode all trees	3895
#6	(insomnia):ti,ab,kw OR (Sleep disturbances)	18458
#7	#1 OR #2	24354
#8	#3 OR #4	13550
#9	#5 OR #6	19036
#10	# 7 AND #8 AND #9	342
**Embass**		
#11	#3 AND #7 AND #10	46
#10	#8 OR #9	134089

#9	sleep disturbances	31218
#8	insomnia	109181
#7	#4 OR #5 OR #6	37608
#6	bzra	109
#5	bzd	2180
#4	benzodiazepines	36637
#3	#1OR #2	180423
#2	chronic obstructive pulmonary disease	137114
#1	copd	126678
**Science of web**		
#	Search Query	Results
1	(TS=(COPD)) OR TS=(chronic obstructive pulmonary disease) and Preprint Citation Index (Exclude – Database)	157755
2	((TS=(BZD)) OR TS=(BZRA)) OR TS=(benzodiazepines) and Preprint Citation Index (Exclude – Database)	49443
3	(TS=(insomnia)) OR TS=(Sleep disturbances) and Preprint Citation Index (Exclude – Database)	102322
4	#1 AND #2 AND #3 and Preprint Citation Index (Exclude – Database)	29

Appendix 3. Quality assessment of cohort studies

Table S2. Quality evaluation of the eligible studies with Newcastle-Ottawa scale (Observational studies).

	Selection				comparability	outcome			score
Study	Representativeness	Selection of non-exposed	Ascertainment of exposure	Outcome not present at start	Comparability on most important factors or other risk factor	Assessment of outcome	Long enough follow- up	Adequacy (completeness) of follow-up	
Su-Jung Chen 2015	1	1	1	1	1*	1	1	1	8
Yi-Hsiang Liao 2020	1	1	1	1	1*	1	1	1	8
Wei-Sheng Chung 2015	0.5	1	1	1	1*	1	1	1	7.5
Nicholas T. Vozoris 2014	1	1	1	1	1*	1	1	1	8
Hao Jiang 2024	1	1	1	1	1*	1	1	1	8

* : One point was deducted because the study did not explicitly report whether patients had comorbid PTSD.

Figure S1.Risk of bias summary （RCTs)

	Random sequence generation (selection bias)	Allocation concealment (selection bias)	Blinding of participants and personnel (performance bias)	Blinding of outcome assessment (detection bias)	Incomplete outcome data (attrition bias)	Selective reporting (reporting bias)	Other bias
Block AJ 1984	?	?	+	?	+	+	+
D Murciano 1993	?	?	+	?	+	+	+
J A Wedzicha 1988	?	+	+	?	+	+	+
Midgren B1989	?	?	+	?	+	+	+
Steens RD 1993	?	?	+	?	+	+	+
Stege G 2010	+	?	+	?	+	+	+
Timms RM 1988	?	?	+	+	+	+	+

Appendix 4. Subgroup analysis

Figure S2. Effect of PAMs on all-cause mortality in COPD patients with comorbid insomnia: A subgroup analysis by drug type.

Study or Subgroup	Experimental Events	Experimental Total	Control Events	Control Total	Weight	Odds Ratio M-H, Random, 95% CI
**13.1.1 BZDs**						
Hao Jiang 2024	4	39	41	544	11.7%	1.40 [0.48, 4.14]
Nicholas T. Vozoris 2014	1398	48915	1533	48915	25.4%	0.91 [0.84, 0.98]
Yi-Hsiang Liao 2020-1	49	2840	21	2840	20.2%	2.36 [1.41, 3.94]
**Subtotal (95% CI)**		**51794**		**52299**	**57.4%**	**1.40 [0.67, 2.92]**
Total events	1451		1595			
Heterogeneity: Tau² = 0.33; Chi² = 13.49, df = 2 (P = 0.001); I² = 85%						
Test for overall effect: Z = 0.90 (P = 0.37)						
**13.1.2 N-BZDs**						
Yi-Hsiang Liao 2020-2	38	2840	21	2840	19.8%	1.82 [1.07, 3.11]
**Subtotal (95% CI)**		**2840**		**2840**	**19.8%**	**1.82 [1.07, 3.11]**
Total events	38		21			
Heterogeneity: Not applicable						
Test for overall effect: Z = 2.19 (P = 0.03)						
**13.1.3 MIX**						
Yi-Hsiang Liao 2020-3	94	2840	50	2840	22.8%	1.91 [1.35, 2.70]
**Subtotal (95% CI)**		**2840**		**2840**	**22.8%**	**1.91 [1.35, 2.70]**
Total events	94		50			
Heterogeneity: Not applicable						
Test for overall effect: Z = 3.65 (P = 0.0003)						
**Total (95% CI)**		**57474**		**57979**	**100.0%**	**1.58 [0.95, 2.61]**
Total events	1583		1666			
Heterogeneity: Tau² = 0.26; Chi² = 34.83, df = 4 (P < 0.00001); I² = 89%						
Test for overall effect: Z = 1.77 (P = 0.08)						
Test for subgroup differences: Chi² = 0.56, df = 2 (P = 0.76), I² = 0%						

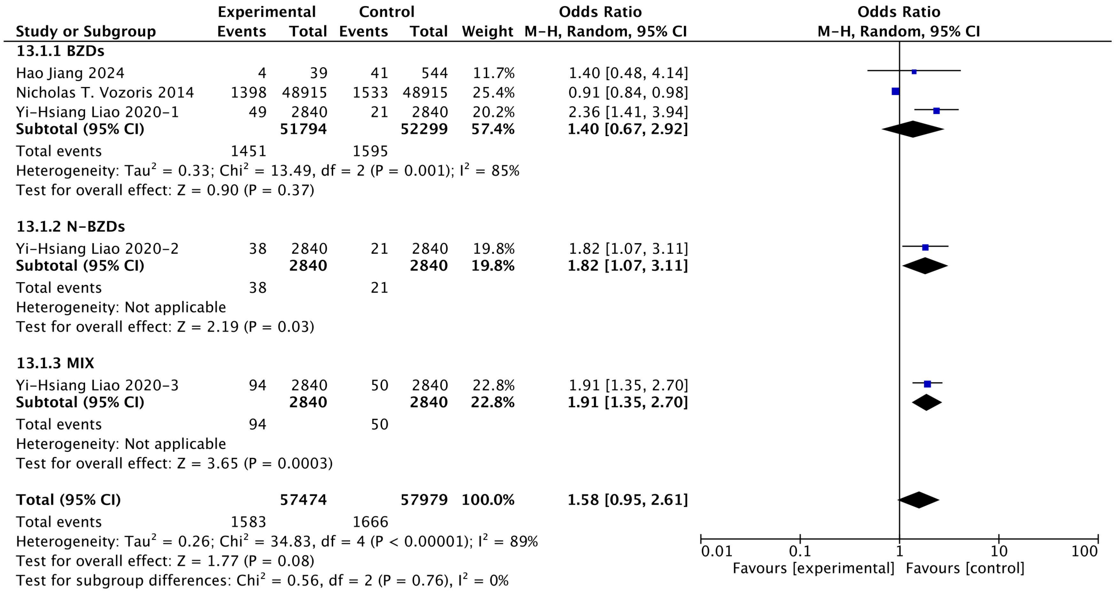

Figure S3. Effect of PAMs on hospitalization for AECOPD in COPD patients with comorbid insomnia: A subgroup analysis by drug type.

Study or Subgroup	Experimental Events	Experimental Total	Control Events	Control Total	Weight	Odds Ratio M-H, Fixed, 95% CI
**14.1.1 BZDs**						
Nicholas T. Vozoris 2014	943	48915	893	48915	93.6%	1.06 [0.96, 1.16]
Yi-Hsiang Liao 2020-1	53	2840	21	2840	2.2%	2.55 [1.54, 4.24]
**Subtotal (95% CI)**		**51755**		**51755**	**95.8%**	**1.09 [1.00, 1.20]**
Total events	996		914			
Heterogeneity: Chi² = 11.20, df = 1 (P = 0.0008); I² = 91%						
Test for overall effect: Z = 1.89 (P = 0.06)						
**14.1.2 N-BZDs**						
Yi-Hsiang Liao 2020-2	44	2840	20	2840	2.1%	2.22 [1.30, 3.77]
**Subtotal (95% CI)**		**2840**		**2840**	**2.1%**	**2.22 [1.30, 3.77]**
Total events	44		20			
Heterogeneity: Not applicable						
Test for overall effect: Z = 2.94 (P = 0.003)						
**14.1.3 MIX**						
Yi-Hsiang Liao 2020-3	53	2840	20	2840	2.1%	2.68 [1.60, 4.50]
**Subtotal (95% CI)**		**2840**		**2840**	**2.1%**	**2.68 [1.60, 4.50]**
Total events	53		20			
Heterogeneity: Not applicable						
Test for overall effect: Z = 3.74 (P = 0.0002)						
**Total (95% CI)**		**57435**		**57435**	**100.0%**	**1.15 [1.05, 1.25]**
Total events	1093		954			
Heterogeneity: Chi² = 28.83, df = 3 (P < 0.00001); I² = 90%						
Test for overall effect: Z = 3.10 (P = 0.002)						
Test for subgroup differences: Chi² = 17.44, df = 2 (P = 0.0002), I² = 88.5%						

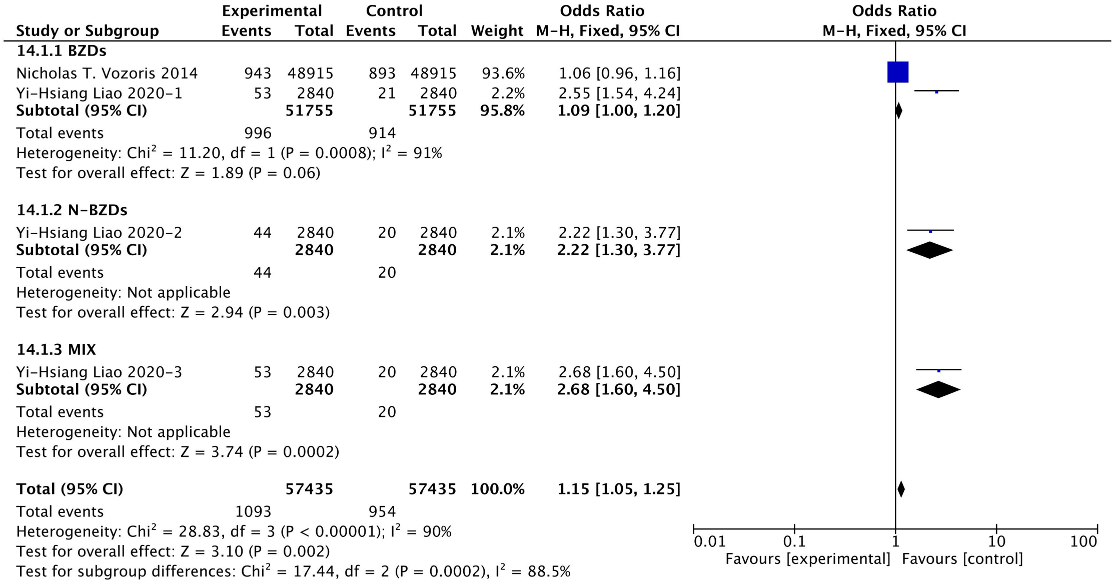

Figure S4. Effect of PAMs on outpatient visits for AECOPD in COPD patients with comorbid insomnia: A subgroup analysis by drug type.

Study or Subgroup	Experimental Events	Experimental Total	Control Events	Control Total	Weight	Odds Ratio M-H, Random, 95% CI
**15.1.1 BZDs**						
Nicholas T. Vozoris 2014	2381	48915	1643	48915	26.1%	1.47 [1.38, 1.57]
Yi-Hsiang Liao 2020-1	361	2840	157	2840	24.8%	2.49 [2.05, 3.02]
**Subtotal (95% CI)**		**51755**		**51755**	**50.9%**	**1.90 [1.13, 3.17]**
Total events	2742		1800			
Heterogeneity: $Tau^2 = 0.13$; $Chi^2 = 25.09$, df = 1 (P < 0.00001); $I^2 = 96\%$						
Test for overall effect: Z = 2.44 (P = 0.01)						
**15.1.2 N-BZDs**						
Yi-Hsiang Liao 2020-2	303	2840	144	2840	24.6%	2.24 [1.82, 2.75]
**Subtotal (95% CI)**		**2840**		**2840**	**24.6%**	**2.24 [1.82, 2.75]**
Total events	303		144			
Heterogeneity: Not applicable						
Test for overall effect: Z = 7.67 (P < 0.00001)						
**15.1.3 MIX**						
Yi-Hsiang Liao 2020-3	355	2840	116	2840	24.5%	3.35 [2.70, 4.17]
**Subtotal (95% CI)**		**2840**		**2840**	**24.5%**	**3.35 [2.70, 4.17]**
Total events	355		116			
Heterogeneity: Not applicable						
Test for overall effect: Z = 10.95 (P < 0.00001)						
**Total (95% CI)**		**57435**		**57435**	**100.0%**	**2.27 [1.52, 3.41]**
Total events	3400		2060			
Heterogeneity: $Tau^2 = 0.16$; $Chi^2 = 78.56$, df = 3 (P < 0.00001); $I^2 = 96\%$						
Test for overall effect: Z = 3.97 (P < 0.0001)						
Test for subgroup differences: $Chi^2 = 8.79$, df = 2 (P = 0.01), $I^2 = 77.2\%$						

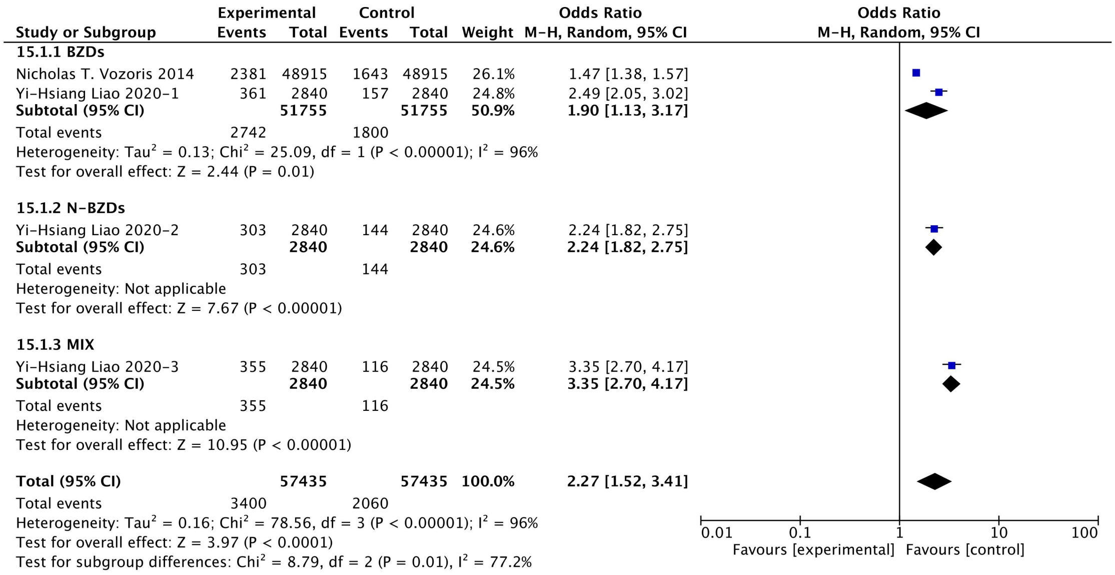

Figure S5. Effect of PAMs on emergency department visits for AECOPD in COPD patients with comorbid insomnia: A subgroup analysis by drug type.

Study or Subgroup	Experimental Events	Experimental Total	Control Events	Control Total	Weight	Odds Ratio M-H, Fixed, 95% CI
**16.1.1 BZDs**						
Nicholas T. Vozoris 2014	694	48915	361	48915	73.1%	1.94 [1.70, 2.20]
Yi-Hsiang Liao 2020-1	94	2840	45	2840	8.9%	2.13 [1.48, 3.04]
**Subtotal (95% CI)**		**51755**		**51755**	**82.1%**	**1.96 [1.73, 2.21]**
Total events	788		406			
Heterogeneity: $Chi^2 = 0.23$, df = 1 (P = 0.63); $I^2 = 0\%$						
Test for overall effect: Z = 10.92 (P < 0.00001)						
**16.1.2 N-BZDs**						
Yi-Hsiang Liao 2020-2	85	2840	40	2840	8.0%	2.16 [1.48, 3.16]
**Subtotal (95% CI)**		**2840**		**2840**	**8.0%**	**2.16 [1.48, 3.16]**
Total events	85		40			
Heterogeneity: Not applicable						
Test for overall effect: Z = 3.98 (P < 0.0001)						
**16.1.3 MIX**						
Yi-Hsiang Liao 2020-3	94	2840	50	2840	9.9%	1.91 [1.35, 2.70]
**Subtotal (95% CI)**		**2840**		**2840**	**9.9%**	**1.91 [1.35, 2.70]**
Total events	94		50			
Heterogeneity: Not applicable						
Test for overall effect: Z = 3.65 (P = 0.0003)						
**Total (95% CI)**		**57435**		**57435**	**100.0%**	**1.97 [1.76, 2.19]**
Total events	967		496			
Heterogeneity: $Chi^2 = 0.50$, df = 3 (P = 0.92); $I^2 = 0\%$						
Test for overall effect: Z = 12.18 (P < 0.00001)						
Test for subgroup differences: $Chi^2 = 0.27$, df = 2 (P = 0.87), $I^2 = 0\%$						

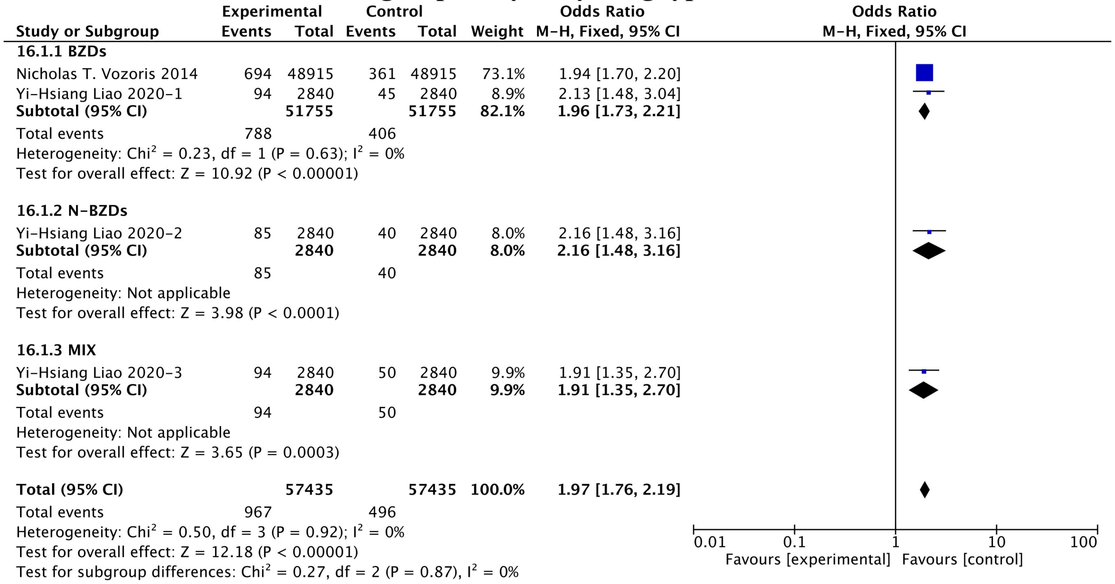

Appendix 5. Leave-one-out sensitivity analysis of the primary outcome

Figure S6. TST

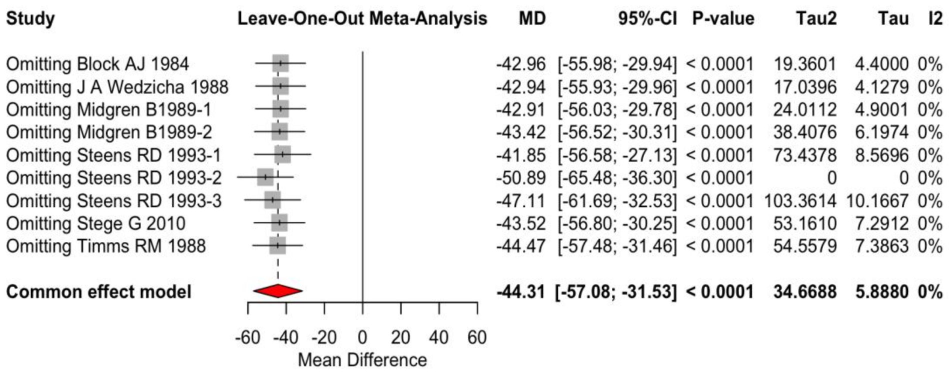

Figure S7. Number of awakenings

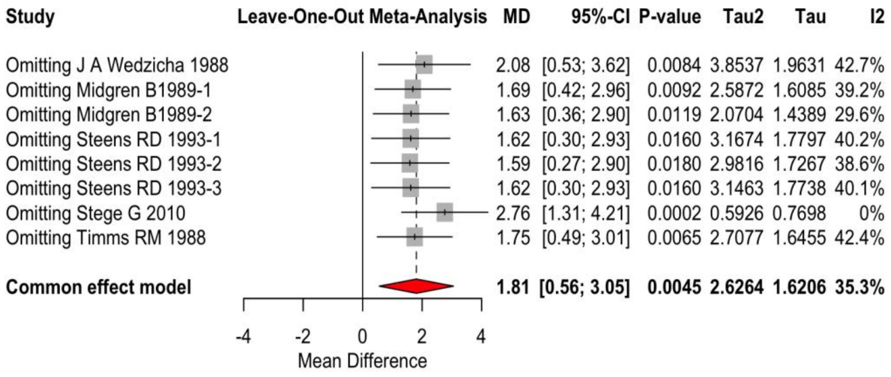

Figure S8. Sleep efficiency

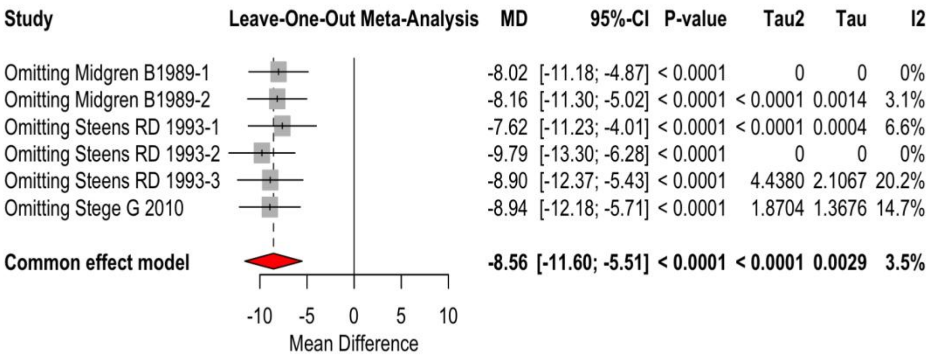

Figure S9. PaO_2

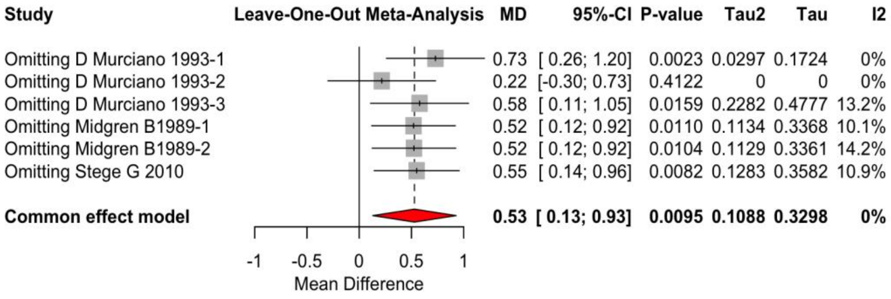

Figure S10. FEV1

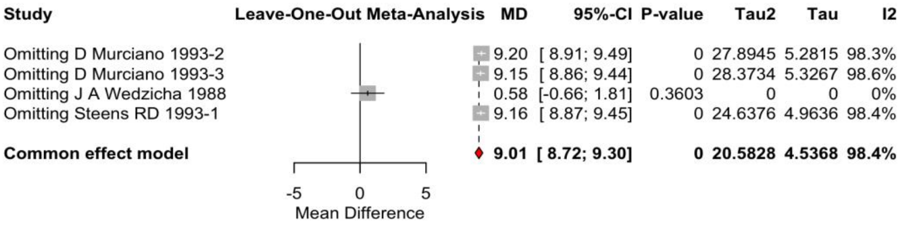

Figure S11. Frequency of apnea events

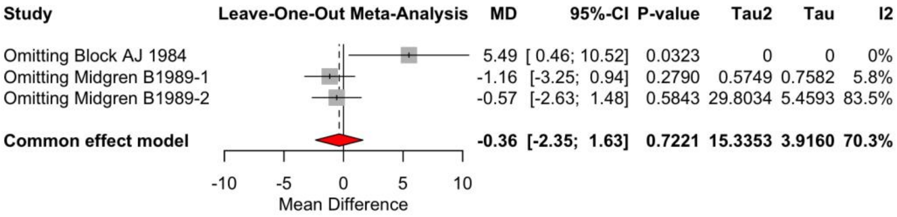

Figure S12. SaO_2

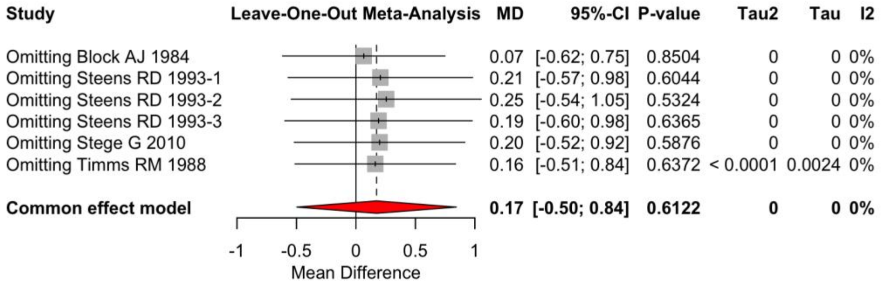

Figure S13. All-cause mortality

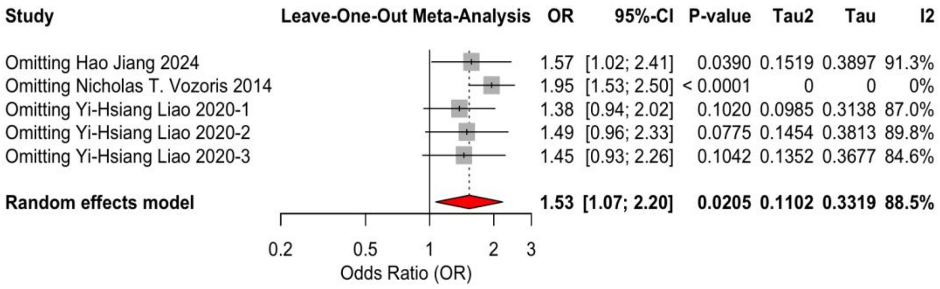

Figure S14. Outpatient for AECOPD

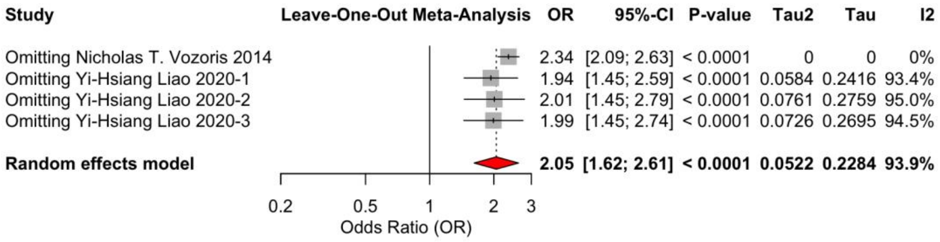

Figure S15. Hospitalization for AECOPD

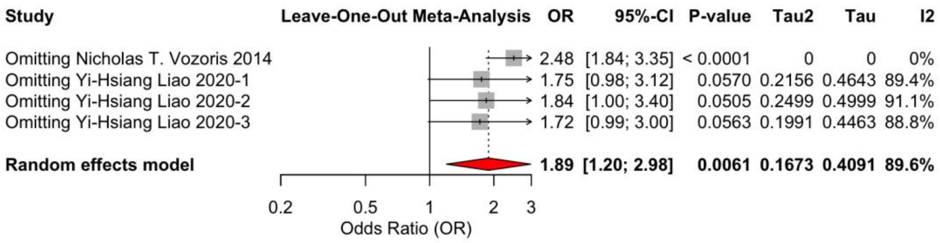

Figure S16. Emergency department for AECOPD

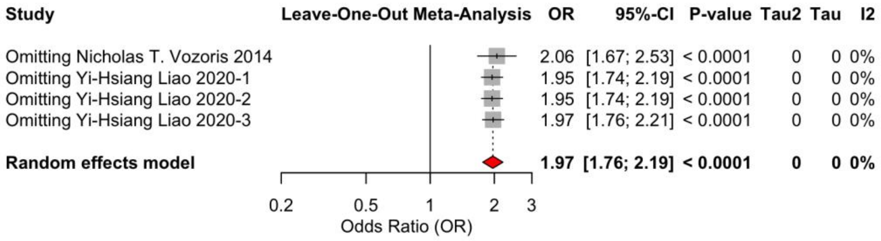

Supplement: Supplementary file 1 [file DataSheet1.pdf]
